# Distinct parasite populations infect individuals identified through passive and active case detection in a region of declining malaria transmission in southern Zambia

**DOI:** 10.1186/s12936-017-1810-3

**Published:** 2017-04-19

**Authors:** Kelly M. Searle, Ben Katowa, Tamaki Kobayashi, Mwiche N. S. Siame, Sungano Mharakurwa, Giovanna Carpi, Douglas E. Norris, Jennifer C. Stevenson, Philip E. Thuma, William J. Moss, for the Southern Africa International Centers of Excellence for Malaria Research

**Affiliations:** 1 Department of Epidemiology, Bloomberg School of Public Health, Johns Hopkins University, Baltimore, MD USA; 2 Macha Research Trust, Choma District, Zambia; 3 Biomedical Research Training Institute, Harare, Zimbabwe; 4 Johns Hopkins Malaria Research Institute, Department of Molecular Microbiology and Immunology, Bloomberg School of Public Health, Johns Hopkins University, Baltimore, MD USA

**Keywords:** Malaria elimination, Molecular barcode, Parasite genetics, Molecular epidemiology, Population genetics, Zambia, Sub-Saharan Africa

## Abstract

**Background:**

Substantial reductions in the burden of malaria have been documented in parts of sub-Saharan Africa, with elimination strategies and goals being formulated in some regions. Within this context, understanding the epidemiology of low-level malaria transmission is crucial to achieving and sustaining elimination. A 24 single-nucleotide-polymorphism *Plasmodium falciparum* molecular barcode was used to characterize parasite populations from infected individuals identified through passive and active case detection in an area approaching malaria elimination in southern Zambia.

**Methods:**

The study was conducted in the catchment area of Macha Hospital in Choma District, Southern Province, Zambia, where the parasite prevalence declined over the past decade, from 9.2% in 2008 to less than 1% in 2013. Parasite haplotypes from actively detected, *P. falciparum*-infected participants enrolled in a serial cross-sectional, community-based cohort study from 2008 to 2013 and from passively detected, *P. falciparum*-infected individuals enrolled at five rural health centres from 2012 to 2015 were compared. Changes in *P. falciparum* genetic relatedness, diversity and complexity were analysed as malaria transmission declined.

**Results:**

Actively detected cases identified in the community were most commonly rapid diagnostic test negative, asymptomatic and had submicroscopic parasitaemia. Phylogenetic reconstruction using concatenated 24 SNP barcode revealed a separation of parasite haplotypes from passively and actively detected infections, consistent with two genetically distinct parasite populations. For passively detected infections identified at health centres, the proportion of detectable polyclonal infections was consistently low in all seasons, in contrast with actively detected infections in which the proportion of polyclonal infections was high. The mean genetic divergence for passively detected infections was 34.5% for the 2012–2013 transmission season, 37.8% for the 2013–2014 season, and 30.8% for the 2014–2015 season. The mean genetic divergence for actively detected infections was 22.3% in the 2008 season and 29.0% in the 2008–2009 season and 9.9% across the 2012–2014 seasons.

**Conclusions:**

Distinct parasite populations were identified among infected individuals identified through active and passive surveillance, suggesting that infected individuals detected through active surveillance may not have contributed substantially to ongoing transmission. As parasite prevalence and diversity within these individuals declined, resource-intensive efforts to identify the chronically infected reservoir may not be necessary to eliminate malaria in this setting.

**Electronic supplementary material:**

The online version of this article (doi:10.1186/s12936-017-1810-3) contains supplementary material, which is available to authorized users.

## Background

Substantial reductions in the burden of malaria have been documented in parts of sub-Saharan Africa [1] and malaria elimination goals have been proposed at regional, national, and subnational levels [2–4]. As areas transition from high or moderate to low malaria transmission and approach elimination, understanding the epidemiology of low-level transmission is crucial to achieving and sustaining elimination [2, 3, 5]. The relative magnitude of infection attributed to locally acquired and imported cases is of interest, specifically the role of the chronically infected, asymptomatic reservoir in maintaining local malaria transmission [6–10]. One way to address this question is to determine the genetic relatedness of parasites between actively detected, predominantly chronically infected, asymptomatic individuals and passively detected, predominantly acutely infected, symptomatic malaria cases [11]. By comparing the genetic overlap between parasites infecting these populations, inferences can be made regarding the relative contribution of the asymptomatic reservoir to symptomatic malaria cases. Understanding the role of these two populations in local malaria transmission dynamics can guide interventions, particularly reactive case detection and focal or mass drug administration strategies that target the chronically infected, asymptomatic reservoir.

A *Plasmodium falciparum* molecular barcode assay consisting of 24 unlinked, single nucleotide polymorphisms (SNPs) has been used to characterize unique genetic signatures and track circulating *P. falciparum* parasite populations [11]. The barcode was developed to elucidate malaria transmission dynamics by tracking the genetic diversity and complexity of the parasite over time and space [12–14], and specifically for use in resource-limited settings as the highest level of technology required is a polymerase chain reaction (PCR) assay [11].

This study was conducted in an area of southern Zambia that experienced a dramatic decrease in malaria transmission over the past decade [15]. The molecular barcode was used to determine the relatedness between parasites over time in infected individuals identified through passive and active surveillance from the same geographic area, as well as changes in parasite genetic complexity and diversity.

## Methods

### Study site and population

The study was conducted in the catchment area of Macha Hospital in Choma District, Southern Province, Zambia, where there is a single rainy season which lasts from November through April, followed by a cool dry season from April until August and a hot dry season from August through November. Malaria transmission peaks during the rainy season [16] and the primary vector is *Anopheles arabiensis* [16, 17]. The hospital catchment area is approximately 1200 km^2^ with roughly 30,000 residents consisting primarily of villagers living in small, scattered homesteads. The prevalence of *P. falciparum* infection declined in this area over the past decade, from 9.2% in 2008 to less than 1% in 2013 [18]. Artemisinin-based combination therapy (ACT) with artemether–lumefantrine was introduced as first-line anti-malarial therapy in Zambia in 2002 [19] and into the study area in 2004. In Zambia, long-lasting insecticide-treated nets (LLINs) are distributed through antenatal care clinics and additional mass distribution campaigns [20]. LLINs were widely distributed in the study area in 2007 [21] and more than 11,000 LLINs were distributed from nine health posts in the catchment area of Macha Hospital in June 2012, with additional LLINs distributed in 2014 according to the Office of the Macha Hospital Environmental Health Technician. ITN ownership was estimated at 83% in the study area in 2013 [15].

### Active community-based malaria surveillance

Satellite images were used to develop a sampling frame for the random selection of households to enroll participants in longitudinal and cross-sectional malaria surveys [21]. The identification and enumeration of households was done manually to delineate household and non-household structures (kraals, schools, and larger structures) [21]. Households selected from the sampling frame were enrolled in either a cross-sectional or longitudinal cohort. Households enrolled in the cross-sectional cohort were surveyed once, whereas households enrolled in the longitudinal cohort were repeatedly surveyed every 2 months. Surveys were conducted each month, alternating between the cross-sectional cohort and the longitudinal cohort, from February 2008 through October 2013. For each study visit, a questionnaire was administered to collect demographic data, history of recent malaria symptoms and treatment, healthcare-seeking behaviour, knowledge of malaria risk and prevention, and long-lasting insecticidal net use. Recent malaria symptoms were defined as having a documented fever greater than 38 °C by tympanic temperature, or reporting a fever and chills within the previous 48 h. A blood sample was collected by finger prick for a rapid diagnostic test (RDT), microscopy and blood was spotted on Whatman 903™ Protein Saver cards. These dried blood spots (DBS) were used to detect *Plasmodium* parasites by a nested PCR as described below and molecular barcoding was attempted for all samples positive for *Plasmodium* parasites by nested PCR (Additional file 1: Figure S1). Participants positive by RDT were offered treatment with artemether-lumefantrine (Coartem^**®**^).

### Passive malaria surveillance at rural health centres

Fourteen rural health centres (RHCs) that serve the catchment population surrounding Macha Hospital sent a weekly text message report of the number of RDTs used, number of positive RDTs, and the number of people treated for malaria to study staff at Macha Research Trust [22]. Five of these RHCs within the study area where active surveillance was conducted were included in a sub-study from 2012 through 2015 that collected DBS from a convenience sample of individuals with a positive RDT and recorded their demographic data. Molecular barcoding was attempted for all samples collected at the RHCs (Additional file 1: Figure S1).

### Laboratory methods

The DBS were stored at −20 °C in individual plastic bags containing desiccant until DNA extraction. DBS collected from February to September 2008 were initially stored at room temperature and subsequently at −20 °C. Parasite DNA was extracted using the Chelex© method from one dried blood spot [18]. Briefly, the DBS were placed in 1.5 mL microcentrifuge tubes, 1 mL of 0.1% weight by volume saponin in 1× phosphate buffered saline (PBS) was added and the mixture was incubated for 10 min at room temperature. The tubes were centrifuged for 2 min at 14,000 rpm, the supernatant discarded and 1 mL of 1× PBS was added. The tubes were again centrifuged for 2 min at 14,000 rpm, the supernatant discarded and 150 μL of 2% weight by volume Chelex© solution and 50 μL of DNase free water were added and the tubes were boiled for 8 min. The tubes were then centrifuged for 1 min at 14,000 rpm and approximately 150 μL of DNA was stored at −20 °C.

Infection was confirmed using a *Plasmodium* multi-species nested PCR assay as previously described [18, 23] targeting a 815 base-pair segment of the mitochondrial cytochrome b gene (*cytb)*. Briefly, in the primary PCR step, 6 μL of DNA extract was added to 0.2 mL tubes containing a 19 μL reaction mix made up of DNase free water and final concentrations of 200 μM dNTPs, 1× ThermoPol PCR buffer (New England Biolabs, Ipswich, MA, USA), 1 μM forward and 1 μM reverse primers and DNA Taq polymerase (New England Biolabs, Ipswich, MA) in 25 μL reaction volume. In the nested PCR step, 3 μL of the primary PCR product was added to 0.2 mL PCR tubes containing 22 μL of reaction mix with DNase free water and the final concentrations of dNTPs, Thermopol buffer, forward and reverse primers, and Taq DNA polymerase in a 25 μL reaction mix as described above. No template controls were included in each experiment and reactions were run in a Techne™ TC-412 thermo cycler (Bibby Scientific Limited, Staffordshire, UK). Amplified product was detected by electrophoresis on 1% agarose gel and viewed under UV light.

The 24 SNP molecular barcode assays were run using a TaqMan protocol at the Macha Research Trust laboratory in Zambia [11]. DNA was extracted from a second dried blood spot for samples positive by nested PCR using the Chelex© method. Due to low parasite DNA concentrations, samples were pre-amplified prior to performing the 24 SNP molecular barcode assay [24]. The pre-amplification step was done by adding 5 μL of DNA from each sample to 10 μL of TaqMan pre-amplification master mix, and 0.2× pooled assay mixture, containing of forward and reverse primers for each of the 24 SNPs as previously described [24].

Pre-amplified samples were diluted 1:20 with TE buffer prior to running the 24 SNP molecular barcoding assay as previously described [24]. For each of the 24 SNP assays, 2.0 μL of pre-amplified sample DNA was added to 10 μL of TaqMan master mix, 7.5 μL distilled water, and 0.5 μL TaqMan commercially available primer and probe assay mixture. For each assay, three known positive controls and two negative, no-template controls were run. Positive controls consisted of DNA samples from *P. falciparum* strains obtained from MR4 (now known as BEI Resources, Manassas, VA, USA) with known haplotypes for all 24 SNPs. Typically, 12 SNP assays were run for five samples at a time with controls on a 96-well plate. The assays were run on the Applied Biosystems StepOnePlus™ (Thermo Scientific, Waltham, MA, USA), and the Roche LightCycler 480™ (Roche Diagnostics Corporation, Indianapolis, IN, USA) real time PCR systems.

Parasites sampled from peripheral blood are those in the haploid intra-erythrocytic stage of their lifecycle. [11]. Thus, SNP calls were made automatically based on allelic discrimination plots using software programs accompanying the real time PCR systems as one of the two alleles or mixed [25, 26]. In cases where SNP calls could not be made automatically, determination was made manually by the study investigators. Otherwise the SNP call was classified as failed. Samples with failed SNP calls were repeated up to three times. If a sample failed on all repeated assays, the SNP was treated as missing data.

### Phylogenetic analysis

Haplotypes, composed of the concatenated 24 SNPs, were aligned using the ClustalW method in MEGA6 software, with mixed calls represented as a third possible allele [13]. Phylogenetic analyses were conducted using the maximum likelihood methods for actively and passively detected malaria infections separately, grouped by malaria transmission season, to explore temporal phylogenetic clustering of haplotypes. Haplotypes for the combined dataset were aligned using the same method and a phylogenetic tree was constructed using the maximum likelihood method.

### Parasite genetic complexity and diversity

Parasite genetic complexity was determined by the number of mixed calls at each of the 24 SNPs. Samples with four or more mixed calls were categorized as polyclonal infections [27]. Temporal trends were graphed and variation in the proportion of polyclonal infections was determined using the Wilcoxon rank-sum test. Samples with more than half missing data were excluded (eight samples from active surveillance and seven samples from passive surveillance).

Parasite genetic diversity was evaluated by determining divergence from the most common barcode for each transmission season stratified by whether the parasite was identified through passive or active surveillance. The most common barcode for each season was determined by allelic frequency at each SNP to determine the nucleotide diversity. A modified ‘SNP π’, was developed to account for missing data and mixed allele calls, and was used to measure the seasonal parasite genetic divergence [14]. Details of the methods for calculating the modified ‘SNP π’ are described in the Additional file 2: Appendix.

### Statistical analyses

Data on age and sex analysed for passively detected cases. Age, sex, symptoms of malaria, and RDT and microscopy results were analysed for actively detected cases. Median ages between actively and passively detected infected individuals and across transmission seasons were compared using a Kruskal–Wallis test. Sex differences between groups and across transmission seasons were compared using a chi^2^ test. RDT and microscopy results across transmission seasons were compared for actively detected cases using a chi^2^ test.

## Results

**Fig. 1:**
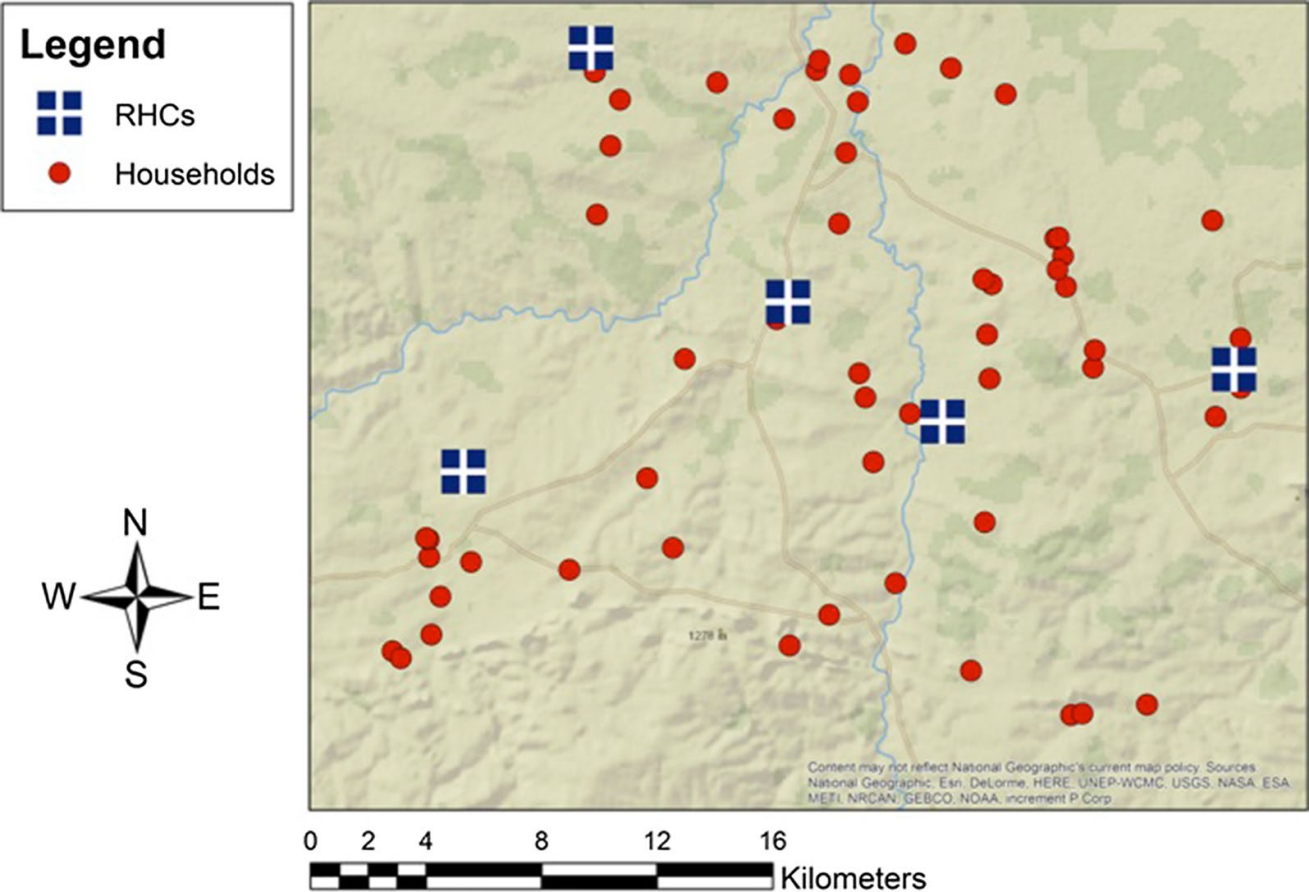
Map of the study area displaying locations of RHCs included in passive malaria surveillance and households of infected individuals included in active community-based malaria surveillance

The 24 SNP molecular barcode was performed on 72 samples from actively detected malaria cases collected from 2008 to 2013 and 46 samples from passively detected malaria cases collected from 2012 to 2015. Households of actively detected cases and the RHC from which the passively detected cases were identified came from the same underlying source population (Fig. 1). The median age was 13 years (IQR = 7–30) for infected individuals identified through passive case detection and 14 years (IQR = 11–21) for those identified through active case detection (p = 0.63) (Table 1). There were no statistically significant differences in sex between the two study populations (p = 0.34), and no statistically significant differences in median age or sex were observed in passively (p = 0.15, p = 0.63 respectively) or actively (p = 0.79, p = 0.96 respectively) detected cases across the malaria transmission seasons (Table 1). While all actively detected cases were identified by PCR, no statistically significant differences were observed in microscopy results across the malaria transmission seasons (p = 0.07), with most infected individuals detected by active case detection negative by microscopy (Table 1). Statistically significant differences were observed in the RDT results across malaria transmission seasons (p = 0.001), with the proportion of RDT positive infections variable, but all seasons had a higher proportion of RDT negative infections among individuals identified through active surveillance (Table 1). Statistically significant differences were observed in the proportion of individuals reporting symptoms of malaria for each season (p = 0.02), with trends in symptomatic infections decreasing from the 2008 season through the 2010–2011 season, then subsequently increasing (Table 1). For both actively and passively detected malaria cases, no identical molecular barcodes were detected and all haplotypes were unique.

**Table 1:** Demographic characteristics of passively and actively detected individuals infected with *Plasmodium falciparum* by malaria transmission season

	Overall	2008^a^	2008–2009	2009–2010	2010–2011	2011–2012	2012–2013	2013–2014	2014–2015
Passively detected cases									
Number	46	–	–	–	–	–	12	25	9
Age in years (median [IQR])	13 [7–30]	–	–	–	–	–	11 [7–30]	18 [9–32]	11 [7–11]
Male (%)	64.4	–	–	–	–	–	63.64	60.00	77.78
Actively detected cases									
Number	72	15	19	13	12	7	6		
Age in years (median [IQR])	14 [11–21]	14 [9–18]	13 [11–18]	17 [11–20]	14.5 [10.5–24]	25 [13–31]	12.5 [6–47]		
Male (%)	55.6	66.7	52.6	53.9	50.0	57.1	50.0		
Symptomatic (%)	34.7	60.0	15.8	23.1	16.7	57.1	66.7		
Microscopy positive (%)	11.1	35.0	7.1	11.1	0.0	25.0	0.0		
RDT positive (%)	20.8	20.0	31.6	23.1	8.3	14.3	16.7		

^a^The 2008 season was truncated as active case detection began in February 2008

### Phylogenetic trees

**Fig. 2:**
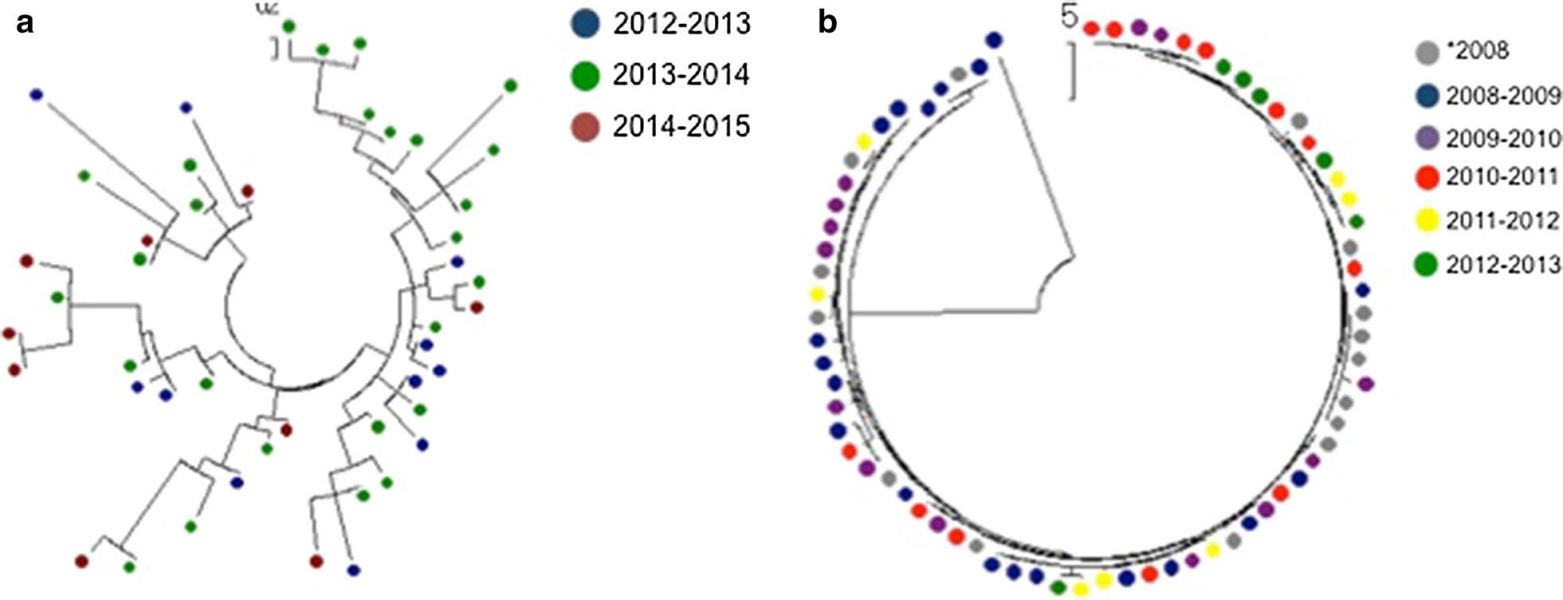
Phylogenetic trees of **a** passively detected infections and **b** actively detected infections, by malaria transmission season

**Fig. 3:**
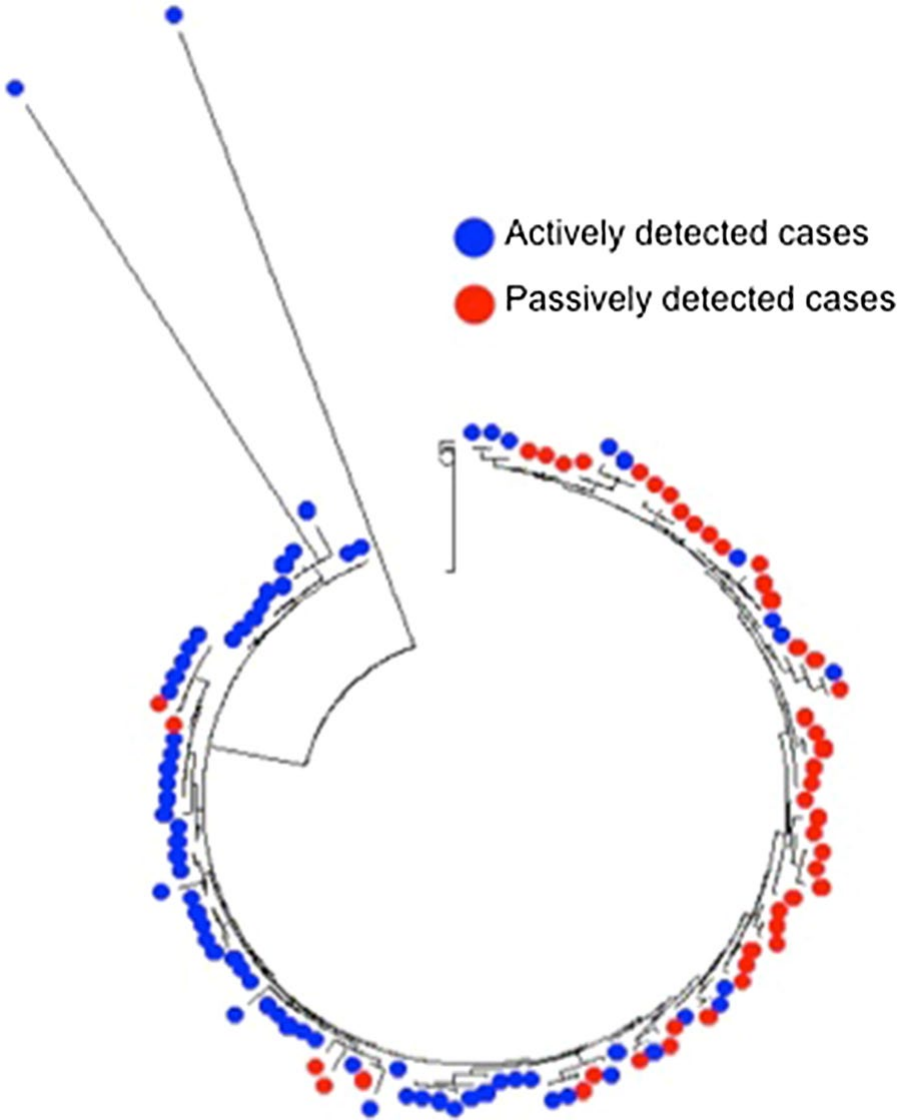
Phylogenetic tree of actively and passively detected infections

The phylogenetic tree showed evidence of clustering of haplotypes from passively detected cases during the 2012–2013 annual malaria transmission season along with some mixing during the other two seasons (Fig. 2a). However, there was no evidence of phylogenetic clustering during the 2013–2014 and 2014–2015 annual malaria transmission seasons (Fig. 2a). For actively detected cases, the phylogenetic tree showed no evidence of phylogenetic clustering during any of the malaria transmission seasons (Fig. 2b). In a combined phylogenetic tree, parasite haplotypes from passively and actively detected cases were largely divergent (Fig. 3), suggesting two genetically distinct parasite populations (Fig. 3).

### Genetic complexity and diversity

**Fig. 4:**
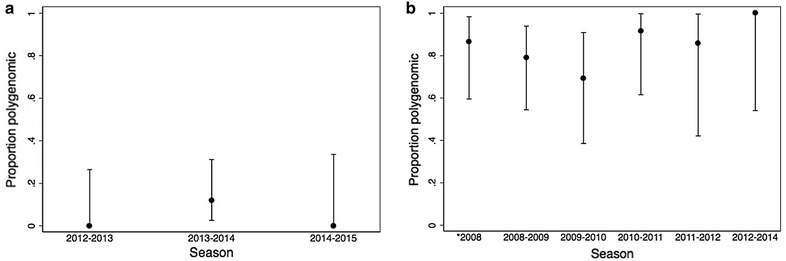
Proportion of polyclonal infections by season for **a** passively detected infections and **b** actively detected infections

Genetic complexity was approximated by calculating the proportion of polyclonal infections in a transmission season. The proportion of polyclonal infections was consistently low for passively detected cases in all seasons, with no polyclonal infections identified during the 2012–2013 and 2014–2015 annual transmission seasons (Table 2; Fig. 4a). In contrast, the proportion of polyclonal infections among actively detected cases was consistently high for all malaria transmission seasons (Fig. 4b). The last two seasons were combined due to the low numbers of cases but all infections were polyclonal (Table 2; Fig. 4b). No seasonal trends in genetic complexity were identified (Fig. 4b).

**Table 2:** Percent of polyclonal infections and genetic divergence for passively and actively detected individuals infected with *Plasmodium falciparum* by malaria transmission season

	Number	Percent polyclonal (95% Confidence interval)	Percent genetic divergence (95% Confidence interval)
Passively detected cases			
2012–2013	12	0 (0–26)	35 (27–42)
2013–2014	25	12 (3–31)	38 (34–42)
2014–2015	9	0 (0–34)	31 (19–43)
Actively detected cases			
2008^a^	15	87 (60–98)	22 (17–28)
2008–2009	19	79 (54–94)	29 (26–32)
2009–2010	13	69 (39–91)	24 (20–28)
2010–2011	12	92 (62–100)	15 (10–19)
2011–2012	7	86 (42–100)	18 (11–26)
2012–2014	6	100 (54–100)	10 (2–17)

^a^The 2008 season was truncated as active case detection began in February 2008

**Fig. 5:**
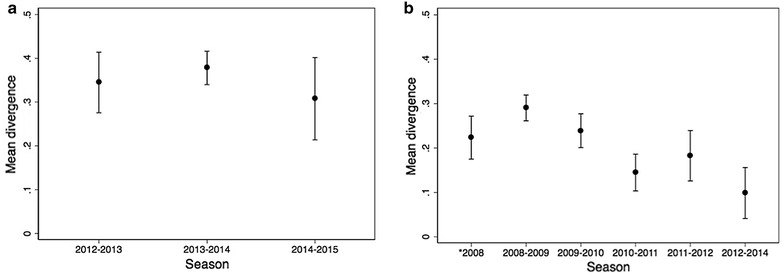
Mean genetic divergence by season for **a** passively detected infections and **b** actively detected infection

The mean genetic divergence for passively detected cases was 34.5% for the 2012–2013 season, 37.8% for the 2013–2014 season, and 30.8% for the 2014–2015 season (Table 2; Fig. 5a). The mean genetic divergence for actively detected cases was 22.3% in the 2008 season and 29.0% in the 2008–2009 season (Table 2; Fig. 5b). This decreased to 9.9% in the combined 2012–2014 seasons (Table 2; Fig. 5b). Overall, the genetic divergence remained high among passively detected cases but decreased for actively detected cases (Fig. 5b).

## Discussion

Distinct parasite populations were found in individuals identified through passive and active surveillance in a region of declining malaria transmission in southern Zambia using a SNP-based molecular barcode for *P. falciparum*. These results suggest that the population of mostly asymptomatic, actively detected infections may not significantly contribute to ongoing transmission in this setting and may not be the primary source of infection leading to clinical malaria cases.

The molecular barcode can be used to document parasite diversity over time by providing information on the number of unique barcodes present each year, the persistence of unique barcodes between years, and the genetic divergence measured by nucleotide diversity within and between years. The molecular barcode determines whether the major allele, minor allele, or a mixture of major and minor alleles is present in the haploid blood stage of the parasite infection for each of the 24 SNPs. The resulting barcode represents the parasite’s haplotype and can be used to compare the genetic relatedness between individual (monoclonal and polyclonal) infections and populations of parasites infecting different communities [28]. Variation arises as a result of genetic recombination and outcrossing during the sexual stage of infection in the mosquito vector when gametocytes combine [13]. SNPs with a mixture of major and minor alleles are referred to as mixed (polyclonal) infections, with higher numbers of mixed infections representing higher genetic complexity. The frequency of mixed infections approximates the level of genetic complexity and provides information about the burden of infection due to genetically unique parasites [27].

Typically, as malaria transmission declines, a genetic bottleneck is created, reducing opportunities for outcrossing in the mosquito midgut and leading to reduced parasite diversity among passively detected, symptomatic malaria cases [13, 28]. This decline in diversity is accompanied by a decline in the complexity of infection, as fewer unique parasites circulate in the population [13, 28]. However, parasite diversity among passively detected, symptomatic infections did not decline in southern Zambia and the complexity of infection was low. As malaria transmission was consistently low between the 2012 and 2015 seasons, the sustained, high parasite diversity in symptomatic infections may be evidence of imported malaria with subsequent local transmission among individuals susceptible to clinical malaria.

Passively detected cases also had fewer complex infections, with a low proportion of polyclonal infections, consistent with recent infection with single parasite clones. In contrast, actively detected cases had more complex infections, with a high proportion of polyclonal infections. Between the 2008 and 2009–2010 transmission seasons there was an indication of a non-statistically significant, decreasing trend in the proportion of polyclonal infections; however, this proportion subsequently increased and remained high, indicating that the actively detected population of mostly asymptomatic and sub-patent infections was composed of many different parasite clones. While transmission declined, these individuals may have harboured parasites acquired over time and maintained at low levels of parasitaemia. Chronic infections have been shown to persist for up to a decade [29–32].

Parasite genetic diversity remained relatively high and constant for passively detected cases throughout the observed malaria transmission seasons. This finding, along with the phylogenetic separation from actively detected cases and the paucity of detectable polyclonal infections, suggests these passively detected cases may represented recent infections with parasites imported into the study area. In contrast, parasite genetic diversity was lower among actively detected cases, consistent with a chronically infected population. The decreasing parasite diversity among the actively detected cases over the study period is consistent with loss of parasites from this chronically infected reservoir as malaria transmission declined.

Previous studies using this genotyping approach were reported from Senegal and Malawi. The molecular barcode was used in Senegal to determine the clonal and epidemic expansion of passively detected clinical *P. falciparum* infections [13]. After enhanced deployment of ITNs and use of RDTs and ACT between 2005 and 2011, reductions in parasite genetic diversity and complexity were observed [13]. Between 2006 and 2013, the molecular barcode was used to determine the decline in malaria transmission among passively detected infections, with re-introduction in 2012 [28]. In Malawi, the molecular barcode was used to determine differences in the complexity of infection between severe clinical malaria cases and cerebral malaria cases among children younger than 5 years of age [27]. Children with cerebral malaria had less complex infections than those with severe malaria [27]. To the authors’ knowledge, this is the first time this molecular barcode was used to determine the genetic relatedness between actively and passively detected infections.

Analyses of spatial hotspots of asymptomatic parasitemia and clinical malaria cases have been previously published from low and moderate transmission settings in Kenya and Mali [32–35]. In the Kenya studies, no spatial overlap was identified between hotspots of asymptomatic malaria infections and clinical malaria infections [33, 34]. This spatial disaggregation supports the hypothesis that the asymptomatic reservoir may not be the major source of infection leading to clinical malaria in low transmission settings. The parasite prevalence in the Macha study area is lower than reported in the Kenya studies. The genetic separation of parasites infecting the asymptomatic and symptomatic populations identified in the current study provides additional support that as areas approach elimination the chronically infected population may not contribute substantially to ongoing transmission and a high proportion of clinical cases are likely due to seasonal importation. This has important implications for the need for resource-intensive efforts to identify and treat the chronically-infected, asymptomatic reservoir through reactive test-and-treat strategies, focal or mass drug administration, or using highly sensitivity diagnostic tests.

There were several major limitations of these analyses. The first was that passive and active case detection did not overlap in time except for one malaria transmission season. However, given the absence of seasonal phylogenetic clustering for both passively and actively detected cases, and because all samples came from the same underlying source population, the combined phylogenetic tree is likely an accurate representation of the phylogenetic relationships between the two parasite populations. Additionally, when constructing the combined phylogenetic tree, samples were indexed by season so within season relationships between the two populations would not be masked. The second limitation was the high number of mixed infections from which single haplotypes were difficult to resolve. To account for this, a third allele was used to indicate mixed infections in calculating the phylogenetic relatedness and mixed infections were accounted for when calculating genetic divergence [13, 14]. The third limitation was that the sample size was relatively small, although this is to be expected in a pre-elimination setting. The fourth limitation was the low level of parasitaemia, also an expected finding in a pre-elimination setting. Pre-amplification of parasite DNA was performed to increase the molecular barcode yield but potentially introduced bias in the allele frequency. However, pre-amplification sites were barcode specific to reduce amplification in non-specific parasite DNA, and all samples were pre-amplified regardless of level of parasitaemia [24]. After pre-amplification, assays with greater than three failures were treated as missing data and samples with over half of data missing were dropped from analyses. However, missing data may have been informative as these samples had low levels of parasitaemia.

## Conclusions

Distinct parasite populations were found among infected individuals identified through active and passive surveillance in a low transmission setting in southern Zambia. The actively detected population represented a chronically infected, reservoir that may not contribute substantially to ongoing transmission. As parasite prevalence and diversity within the actively detected cases declined over time, resource-intensive efforts to identify and treat the chronically infected reservoir through reactive case detection, or focal or mass drug administration, may not be necessary to eliminate malaria in this setting.

## Additional files

**Additional file 1.** Flow diagram of sample selection.

**Additional file 2.** The modified ‘SNP π’ used to account for missing data and mixed allele calls was calculated as follows.
