# Supplementary figures and images for: Distinct parasite populations infect individuals identified through passive and active case detection in a region of declining malaria transmission in southern Zambia
Source: Malar J. 2017 Apr 19;16:154. doi: 10.1186/s12936-017-1810-3 (PMC5395854; doi:10.1186/s12936-017-1810-3)

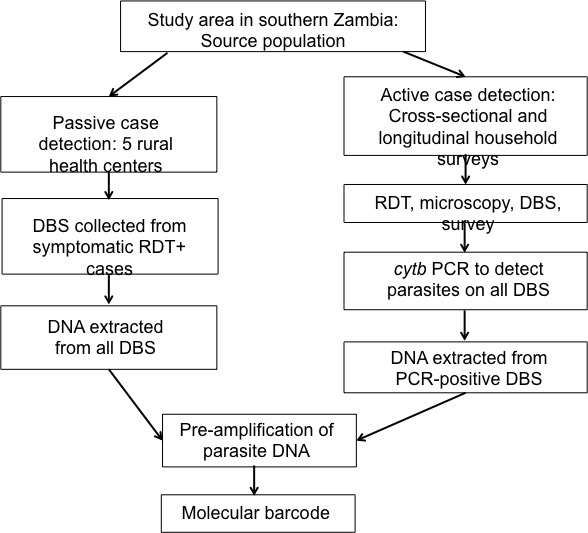

Supplement: Supplementary file 1 — Additional file 1. Flow diagram of sample selection. [file 12936_2017_1810_MOESM1_ESM.jpg]
