# Supplementary material for: Distinct parasite populations infect individuals identified through passive and active case detection in a region of declining malaria transmission in southern Zambia
Source: Malar J. 2017 Apr 19;16:154. doi: 10.1186/s12936-017-1810-3 (PMC5395854; doi:10.1186/s12936-017-1810-3)
Supplement: Supplementary file 2 — Additional file 2. The modified ‘SNP π’ used to account for missing data and mixed allele calls was calculated as follows. [file 12936_2017_1810_MOESM2_ESM.docx]

## Appendix

The modified ‘SNP π’ used to account for missing data and mixed allele calls was calculated as follows:

1. The most common barcode for each season was determined based on allelic frequency for each of the 24 sites
2. For each sample, the maximum agreement that could be achieved was calculated for all non-missing 24 sites. SNP calls of only allele 1 or allele 2 were given a score of 1 and mixed calls were given a score of 0.5.
3. For each sample, the maximum agreement for all non-missing alleles was summed to give a total potential agreement score. For example, a sample with no missing data and no mixed calls would have a total potential agreement score of 24.
4. The agreement between individual samples and the most common barcode was calculated. For each of the 24 SNPs, each sample received a score of 1 if the allele was the same as the most common barcode and a score of zero otherwise. When the most common allele at a given SNP was a mixed call, the barcode would receive a score of 0.5 for that SNP. If a barcode had a mixed allele assignment at a SNP for which the most common barcode was not mixed, the sample also received a score of 0.5 for that SNP.
5. The agreement across all non-missing sites was summed to create a total observed agreement score.
6. The proportion of agreement for each sample was calculated as the total observed agreement score divided by the total potential agreement score.
7. The proportion of divergence was calculated as 1 minus the percent agreement for each sample.

For each season, the mean divergence was calculated to allow for comparisons across seasons.
